# Novel Triterpenoids from *Cassia fistula* Stem Bark Depreciates STZ-Induced Detrimental Changes in IRS-1/Akt-Mediated Insulin Signaling Mechanisms in Type-1 Diabetic Rats

**DOI:** 10.3390/molecules26226812

**Published:** 2021-11-11

**Authors:** Sabapathy Indu, Periyasamy Vijayalakshmi, Jayaraman Selvaraj, Manikkam Rajalakshmi

**Affiliations:** 1 DBT-BIF Centre, PG & Research Department of Biotechnology & Bioinformatics, Holy Cross College (Autonomous), Bharathidasan University, Trichy 620002, Tamil Nadu, India; sabaindhu2010@gmail.com (S.I.); pvijibi@gmail.com (P.V.); 2 Department of Biochemistry, Saveetha Institute of Medical and Technical Sciences, Saveetha Dental College and Hospitals, Saveetha University, Chennai 600020, Tamil Nadu, India; jselvaendo@gmail.com

**Keywords:** *Cassia fistula*, novel triterpenoids, diabetes mellitus, insulin signaling, in vivo, in silico

## Abstract

Here, we identified the mechanisms of action of antidiabetic activity of novel compounds isolated from *Cassia fistula* stem bark in STZ-diabetic animals. Novel triterpenoid compounds (C1, C2 and C3) were treated to STZ-administered diabetic animals at a concentration of 20mg/kg body weight orally for 60 days to assess their effects on plasma glucose, plasma insulin/C-peptide, serum lipid markers and the enzymes of carbohydrate metabolism, glucose oxidation and insulin signaling molecules. Oral administration of novel triterpenoid compounds to STZ-diabetic animals significantly decreased (*p* < 0.05) the plasma glucose concentration on the 7th, 15th, 30th, 45th and 60th daysin a duration-dependent manner (*p* < 0.05). Plasma insulin (*p* < 0.0001)/C-peptide (*p* < 0.0006), tissue glycogen (*p* < 0.0034), glycogen phosphorylase (*p* < 0.005), glucose 6-phosphatase (*p* < 0.0001) and lipid markers were significantly increased (*p* < 0.0001) in diabetic rats, whereas glucokinase (*p* < 0.0047), glycogen synthase (*p* < 0.003), glucose oxidation (*p* < 0.001), GLUT4 mRNA (*p* < 0.0463), GLUT4 protein (*p* < 0.0475) and the insulin-signaling molecules IR mRNA (*p* < 0.0195), IR protein (*p* < 0.0001), IRS-1 mRNA (*p* < 0.0478), p-IRS-1^Tyr612^ (*p* < 0.0185), Akt mRNA (*p* < 0.0394), p–Akt^Ser473^ (*p* < 0.0162), GLUT4 mRNA (*p* < 0.0463) and GLUT4 (*p* < 0.0475) were decreased in the gastrocnemius muscle. In silico analysis of C1–C3 with IRK and PPAR-γ protein coincided with in vivo findings. C1–C3 possessed promising antidiabetic activity by regulating insulin signaling mechanisms and carbohydrate metabolic enzymes.

## 1. Introduction

Skeletal muscle is the key site for insulin-activated glucose consumption in vivo. Skeletal muscle insulin resistance is a primary and important phenomenon for the progression of type-1 diabetes mellitus (T1DM) [1,2]. About 85% of the whole-body insulin-initiated uptake of glucose in skeletal muscle is driven by the translocation of glucose transporter, GLUT4, from the cytoplasmic region to the plasma membrane [3]. Many intracellular signaling cascades are involved in the translocation process, such as provocation of insulin receptor substrate (IRS) molecules, phosphatidylinositol-3-kinase (PI3K) and protein kinase B or Akt [4]. Any deficit in the absorption of glucose in the skeletal muscles contributes to insulin resistance in the entire body [5]. Though numerous antidiabetic drugs are clinically used in the treatment of T1DM, their unfavorable effects, including anemia, pancreatitis, weight gain, bone fracture and cancer, limits the continuing use of these drugs in diabetic patients [6,7]. This in turn leads to increased predisposal of these patients to lethal diabetic difficulties with cardiovascular disease and stroke. For this reason, the progress of harmless potential drugs to treat T1DMis extremely necessary.

Herbal compounds have been utilized since early times to treat T1DM [8]. About 90% of the population in developing countries relies on traditional medicines for its primary health care [9]. In this context, we identified three novel tetracyclic triterpenoids (cpd-1(2879/CHE/2012), cpd-2 (2880/CHE/2012 and cpd-3(2881/CHE/2012)) and isolated them from the ethyl acetate extract of *Cassia fistula* (*C. fistula*) stem bark.

Tetracyclic triterpenoids reported in medicinal plants are widely used in managing the ailments of diabetes [10]. They have a broad range of other bioactivities, including antioxidative properties in the nitric oxide pathway, improvement of insulin sensitivity, stimulation of glucose uptake, regulation of hepatic gluconeogenesis, and antidiabetic, antihyperlipidemic and hepatoprotective effects [11,12,13,14,15]. Tetracyclic triterpenoids stimulate the expression of GLUT4 mRNA and IRS-1 in 3T3-L1 adipocytes [12], strongly activate AMPK, increase β-oxidation and glucose uptake with increasing GLUT4 translocation in L6 myotubes and 3T3-L1 adipocytes and increase the tyrosine phosphorylation level in IRS-1 in FL83B and C2C12 cells [16,17,18]. The referenced studies demonstrated the molecular mechanisms of tetracyclic triterpenoids in glucose metabolism.

Previous findings from our laboratory showed that novel catechin isolated from *C. fistula* stem bark possessed hypolipidemic and hypoglycemic activity in streptozotocin (STZ)-administered diabetic animals. It also enhanced glucose oxidation through GLUT4 in the skeletal muscle [19]. As an extension of that study, the present investigation was focused on the role of three novel tetracyclic triterpenoids in the IRS-1/Akt-mediated insulin signaling mechanisms in the gastrocnemius muscle of STZ-induced diabetic rats. The interactions of these triterpenoids with IRS-I and peroxisome proliferator-activated receptor gamma (PPAR-γ) proteins were analyzed through an in silico docking study using Schrodinger.

## 2. Results

### 2.1. Structure of Novel Tetracyclic Triterpenoids

**Figure 1:**
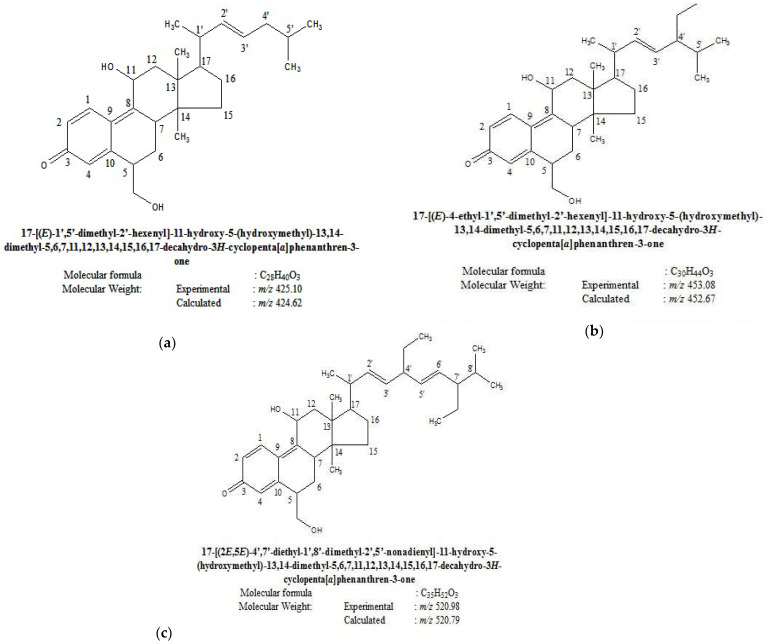
Structures of the novel compounds (**a**) 2879/CHE/2012(cpd-1); (**b**) 2880/CHE/2012(cpd-2); (**c**) 2881/CHE/2012(cpd-3).

As the compounds gave a single spot in TLC, the purity was 99.99%. The spectral data obtained through FT-IR, ESIMS, ^1^HNMR and ^13^C NMR (Supplementary Material, Figures S1–S3) and the structures, molecular weight and molecular formula identified from the spectral data are presented in Figure 1a–c. The compounds were identified as novel tetracyclic triterpenoids and filed for Indian patenting (2879/CHE/2012, 2880/CHE/2012 and 2881/CHE/2012), published in the Patent Office Journal 30 November 2012.

### 2.2. Effect of Novel Compounds on Plasma Glucose Levels

**Figure 2:**
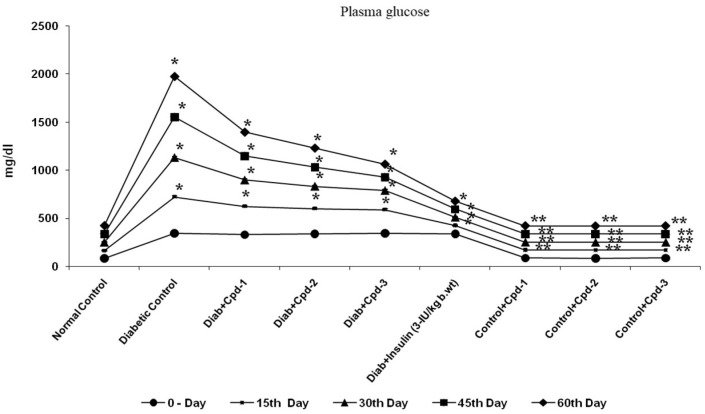
Effect of novel triterpenoids on plasma glucose in STZ-induced diabetic rats compared with the effect of insulin. Each bar represents Mean ± SEM of 6 animals. Significance at *p* < 0.05; *, compared with control; **, compared with diabetic control.

In STZ-administered diabetic animals, the plasma glucose concentration was significantly raised compared to that in control animals. Oral administration of the triterpenoid compounds (20 mg/kg body weight) to diabetic rats reduced the plasma glucose concentration significantly on the 7th, 15th, 30th, 45th and 60th days in a duration-dependent manner. Furthermore, 60 days of treatment effectively reduced the plasma glucose concentration to a level equal to that of the normal control rats and insulin-treated diabetic rats (Figure 2). Hence, 60-day-treated rats were considered for further biochemical, mRNA and protein expression analysis.

### 2.3. Effect of Novel Compounds on Plasma Insulin and C-Peptide Concentration

**Figure 3:**
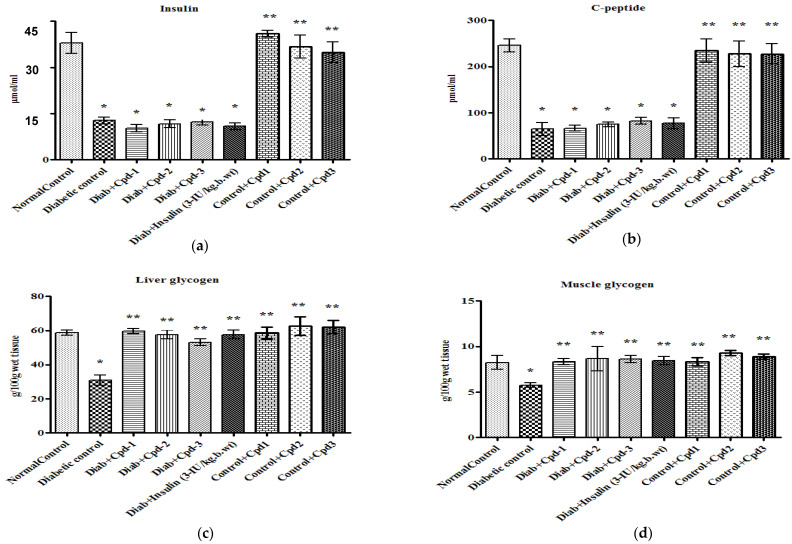
Effect of novel triterpenoids on (**a**) plasma insulin; (**b**) C-peptide; (**c**) liver glycogen; (**d**) muscle glycogen in STZ-induced diabetic rats compared with the effect of insulin. Each bar represents mean ± SEM of 6 animals. Significance at *p* < 0.05; *, compared with control; **, compared with diabetic control.

Plasma insulin and C-peptide concentrations were decreased significantly in STZ-administered diabetic animals. Diabetic rats treated with novel triterpenoids (C1–C3) and insulin did not have increased plasma insulin and C-peptide concentrations compared to the control (Figure 3a,b).

### 2.4. Effect of Novel Compounds on Liver and Muscle Glycogen Concentration

Tissue glycogen content in the liver and skeletal muscle of control and diabetic animals are shown in Figure 3c,d. STZ administration diminished the glycogen concentration in both the skeletal muscle and liver, while treatment with the novel compounds significantly raised the glycogen concentration to the same level as in insulin-treated rats.

### 2.5. Effect of Novel Compounds on Glucose Metabolic (Glucokinase) and Gluconeogenic Enzymes (Glucose 6-Phosphatase) Activity

**Figure 4:**
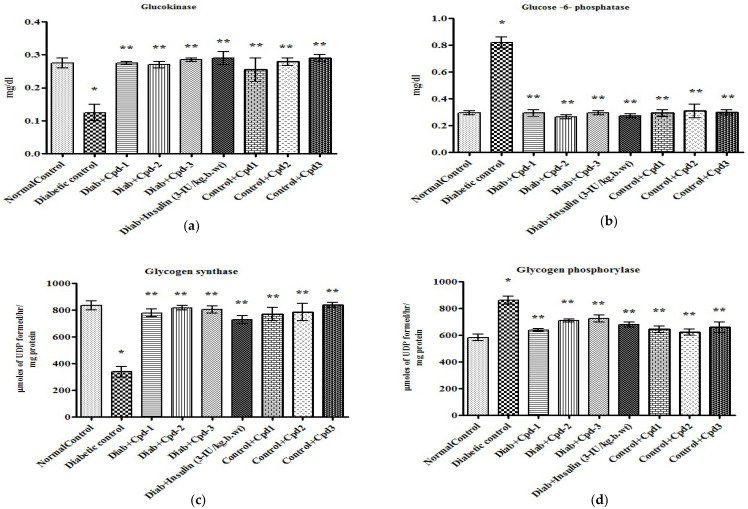
Effect of novel triterpenoids on (**a**) glucokinase; (**b**) glucose 6-phosphatase; (**c**) glycogen synthase; (**d**) glycogen phosphorylase in STZ-induced diabetic rats compared with the effect of insulin. Each bar represents mean ± SEM of 6 animals. Significance at *p* < 0.05; *, compared with control; **, compared with diabetic control.

Figure 4a,b illustrates the effect of novel compounds on glucokinase and glucose 6-phosphatase enzyme levels. Glucokinase enzyme activity was significantly reduced in STZ-administered diabetic animals (Figure 4a), while the activity of glucose-6 phosphatase was significantly raised (Figure 4b). Treatment with the novel compounds to the diabetic animals for 60 days normalized the altered levels of these enzyme activities to a level equal to that in insulin-treated rats.

### 2.6. Effect of Novel Compounds on the Activity of Glycogen Metabolizing Enzymes

The activity of the enzyme glycogen synthase, which is involved in glycogenesis, was decreased significantly in STZ-administered diabetic animals (Figure 4c). The activity of glycogen phosphorylase, which is involved in glycogenolysis, was significantly raised in diabetic rats (Figure 4d). These activities were partially restored by compound administration compared to normal control rats and insulin-treated diabetic rats.

### 2.7. Effect of Novel Compounds on Lipid Markers

**Figure 5:**
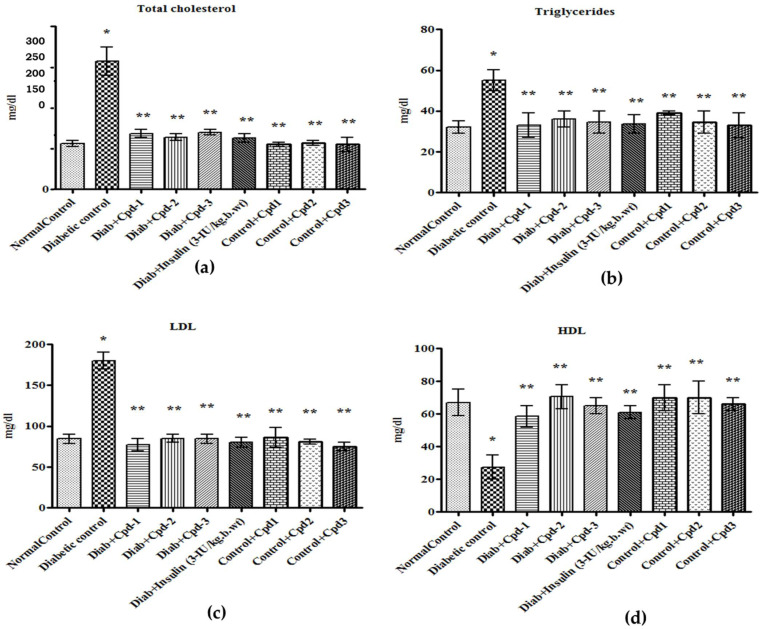
Effect of novel triterpenoids on (**a**) TC; (**b**) TG; (**c**) LDL; (**d**) HDL in STZ-induced diabetic rats compared with the effect of insulin. Each bar represents mean ± SEM of 6 animals. Significance at *p* < 0.05; *, compared with control; **, compared with diabetic control.

Diabetic animals exhibited a significant increase in the levels of serum total cholesterol (TC), triglyceride (TG) and low-density lipoprotein cholesterol (LDL) and a significant reduction in the level of high-density lipoprotein cholesterol (HDL) (Figure 5a–d). Oral administration of novel compounds normalized these conditions to those of the control and insulin-treated diabetic animals.

### 2.8. Effect of Novel Compounds on Glucose Oxidation in Skeletal Muscle

**Figure 6:**
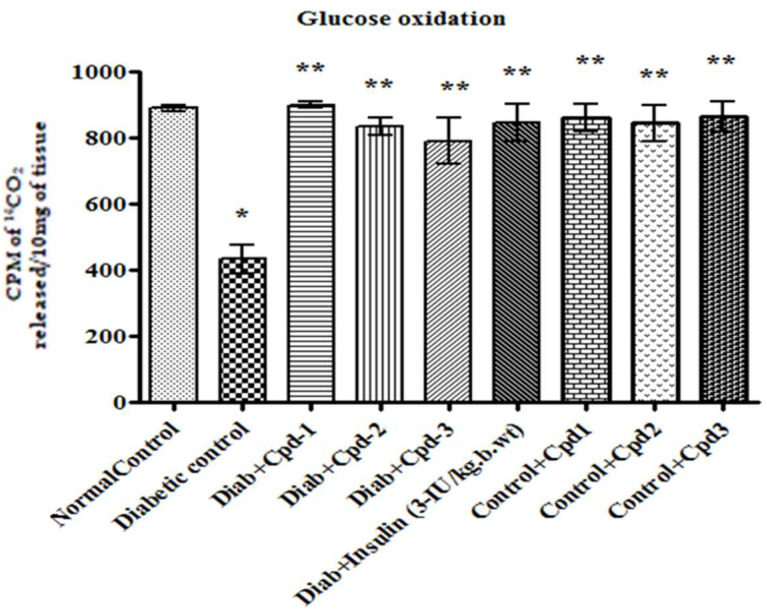
Effect of novel triterpenoids on glucose oxidation in STZ-induced diabetic rats compared with the effect of insulin. Each bar represents mean ± SEM of 6 animals. Significance at *p* < 0.05; *, compared with control; **, compared with diabetic control.

In diabetic animals, oxidation of glucose was significantly decreased compared to control (Figure 6). However, oral administration of novel triterpenoids increased glucose oxidation to a level similar to that in insulin-treated diabetic animals.

### 2.9. Effect of Novel Compounds on IR and IRS-1mRNA and Protein Expression in Skeletal Muscle

**Figure 7:**
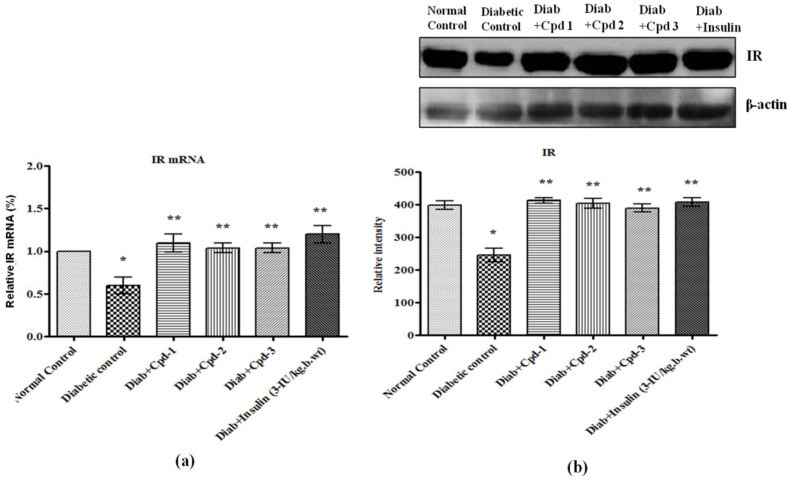
Effect of novel triterpenoids on (**a**) IR mRNA; (**b**) IR protein and its expression in skeletal muscle of STZ-induced diabetic rats compared with the effect of insulin. The expression of mRNA and proteins was assessed by real-time PCR and Western blotting. β-actin was used as a loading control. Each bar represents mean ± SEM of 3 observations representing 6 animals. Significance at *p* < 0.05; *, compared with control, **, compared with diabetic control.

**Figure 8:**
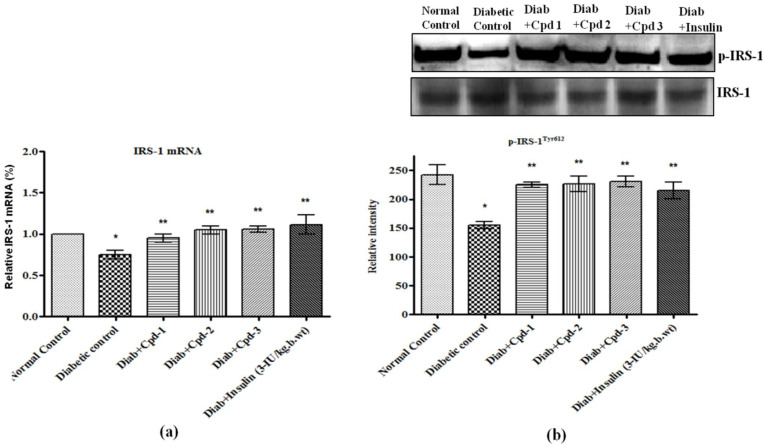
Effect of novel triterpenoids on (**a**) IRS-1 mRNA; (**b**) p-IRS-1 protein and expression in skeletal muscle of STZ-induced diabetic rats compared with the effect of insulin. The expression of mRNA and proteins was assessed by real-time PCR and Western blotting. β-actin was used as a loading control. Each bar represents mean ± SEM of 3 observations representing 6 animals. Significance at *p* < 0.05; *, compared with control, **, compared with diabetic control.

Insulin receptor (IR), IRS-1 mRNA and their phosphorylation (p-IRS-1^612^) were decreased significantly in STZ-administered diabetic animals, but novel compound treatment significantly raised the same (Figure 7a,b and Figure 8a,b).

### 2.10. Effect of Novel Compounds on Akt mRNA andProtein Expression in Skeletal Muscle

**Figure 9:**
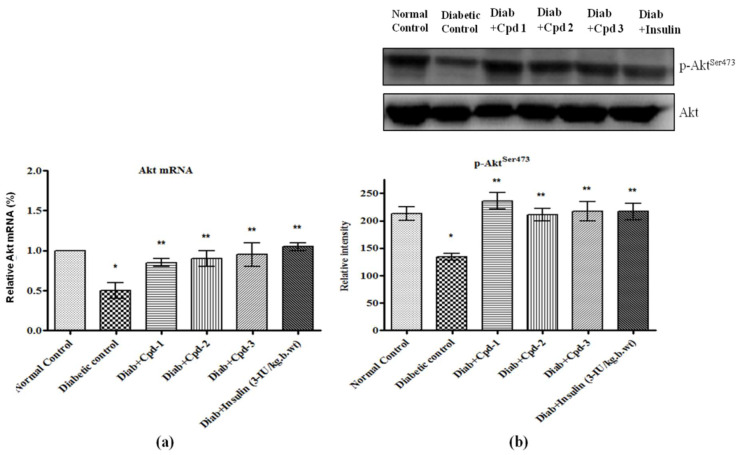
Effect of novel triterpenoids on (**a**) Akt mRNA; (**b**) p-Akt protein and expression in skeletal muscle of STZ-induced diabetic rats compared with the effect of insulin. The expression of mRNA and proteins was assessed by real-time PCR and Western blotting. β-actin was used as a loading control. Each bar represents mean ± SEM of 3 observations representing 6 animals. Significance at *p* < 0.05; *, compared with control; **, compared with diabetic control.

Akt mRNA and its serine phosphorylation (p–Akt^Ser473^) were reduced significantly in diabetic animals (Figure 9a,b). Administration of compounds to the diabetic animals increased the mRNA and protein levels of Akt to levels equal to those in insulin-treated diabetic rats.

### 2.11. Effect of Novel Compounds on GLUT4 mRNA and Protein Expression in Skeletal Muscle

**Figure 10:**
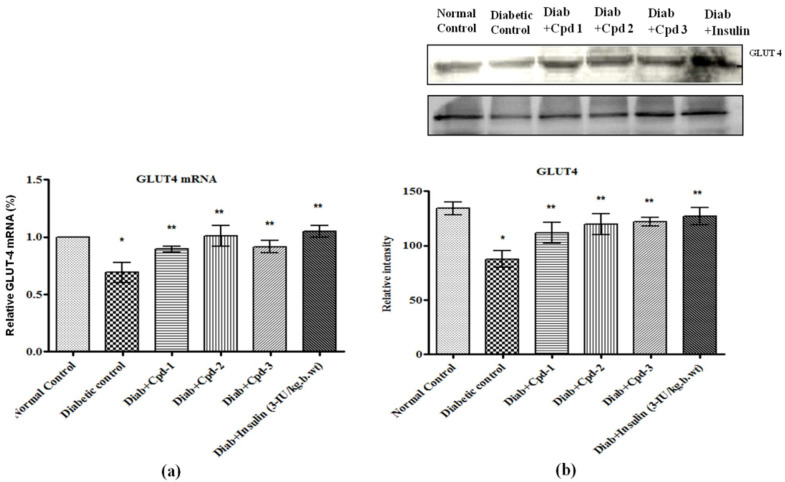
Effect of novel triterpenoids on (**a**) GLUT4 mRNA; (**b**) protein expression in skeletal muscle of STZ-induced diabetic rats compared with the effect of insulin. The expression of mRNA and proteins was assessed by real-time PCR and Western blotting. β-actin was used as a loading control. Each bar represents mean ± SEM of 3 observations representing 6 animals. Significance at *p* < 0.05; *, compared with control, **, compared with diabetic control.

GLUT4 mRNA and protein expression were decreased significantly in the diabetic animals, but compound treatment significantly raised the same (Figure 10a,b). Oral administration of novel triterpenoids restored the mRNA and protein levels of GLUT4 to be equal to those in control and insulin-treated diabetic rats.

### 2.12. The Binding Mode of Compounds with IRK and PPAR-γ

**Figure 11:**
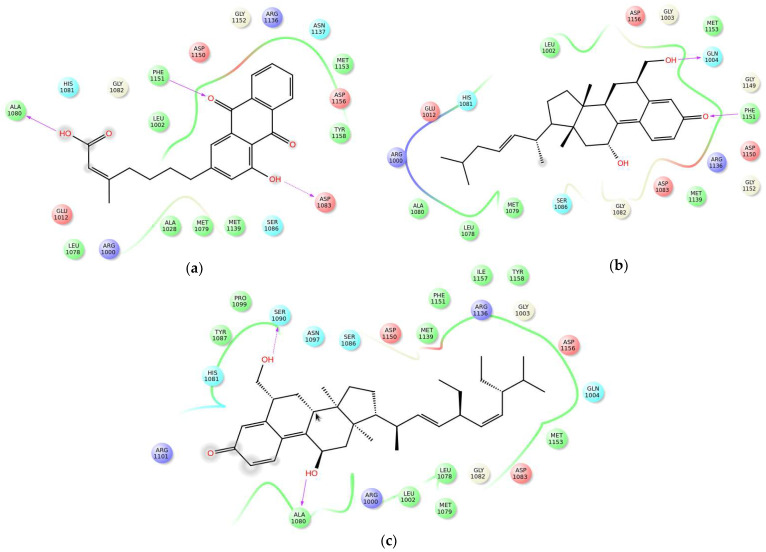

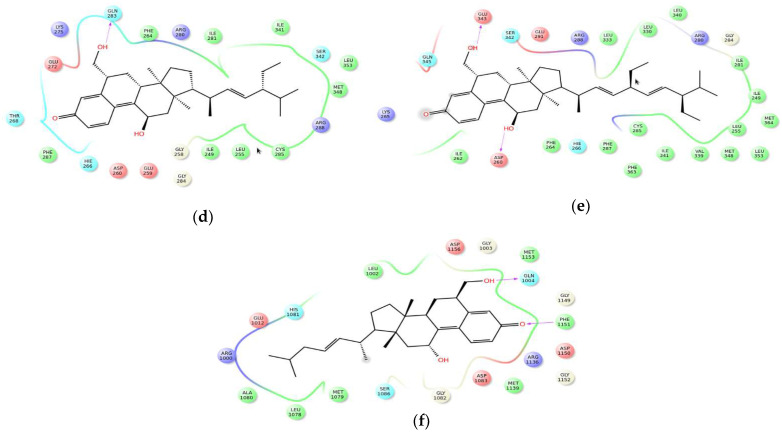
Molecular interaction poses of the 3 compounds with IRK and PPAR-γ: (**a**) the interaction between IRK and compound **1**; (**b**) the interaction between IRK and compound **2**; (**c**) the interaction between IRK and compound **3**; (**d**) the interaction between PPAR-γ and compound **1**; (**e**) the interaction between PPAR-γ and compound **2**; (**f**) the interaction between PPAR-γ and compound **3**.

Docking analysis of the novel compounds in the binding sites of IRK and PPAR-γ was performed with Glide XPin Schrodinger software. The target proteins were docked with selected compounds by the least values of docking score and energies. The top binding affinities of the three compounds with IRK and PPAR-γ binding sites are shown in Figure 11a–c and Figure 11d–f, respectively. Their corresponding docking scores, energy values and H bond details are listed in Table 1.

## 3. Discussion

In the present study, spectral analysis of three compounds from the ethyl acetate extract of C. fistula stem bark revealed that they were novel triterpenoid compounds. Terpenoids have been reported as antidiabetic agents [20]. The skeletal muscle is the largest postprandial site for glucose disposal, and the most important mechanism for maintaining glucose homeostasis is insulin-stimulated glucose uptake in this tissue. The reduced glucose concentration in the compound-administered animals may have resulted from increased glucose oxidation by enhanced GLUT4 proteins. Insulin release from pancreatic β-cells, impaired glucose absorption in the intestine, induced glycogenesis in the liver or insulin-like activity of the compounds could also have accomplished this reduction in blood glucose.

The pancreatic β-cell has the ability to respond to small changes in plasma glucose levels, thereby maintaining the amount of blood glucose within a very narrow range. Progressive degradation of pancreatic β-cells causing reduced insulin production and subsequent hyperglycemia is observed in all types of T1DM. Similar findings could be seen in STZ-administered animals, which were also attributable to the progressive degradation of pancreatic β-cells. Since β-cells release equimolar quantities of insulin and C-peptide, it is very much clear that the level of C-peptide decreased proportionally as well. In the present study, the oral administration of novel compounds to diabetic rats did not lead to any change in the insulin and C-peptide concentration, which suggests that the novel triterpenoids did not have any influence over secretion of pancreatic insulin. Our previous study on catechin isolated from methanol extract of the stem bark of C. fistula showed the same kind of impact in the pancreases of STZ-induced diabetic rats, and we concluded that they possessed insulin mimetic activity [21]. Other studies investigated using diabetes rat models with pentacyclic triterpenoids, oleanolic acid, gymnemic acid IV, ursolic acid and ficusonolide exhibited pharmacological properties including improved insulin secretion and signaling via important transduction pathways, restored β-cell function, reduced hyperglycemia and increased glucose uptake in skeletal muscles [22,23,24]. Triterpenoid saponins from Primula denticulate were reported to potentially possess glucose-lowering properties in STZ-induced diabetic rats [25]. Bassic acid, an unsaturated triterpene acid, was reported to increase glucose absorption and glycogen synthesis and increase plasma insulin levels in alloxan-diabetic rats [26]. Hence, the novel compounds could possess insulin-mimetic activity.

The level of serum lipids in T1DM is typically elevated, which is a risk factor for coronary heart disease [27]. In the present study, STZ-administered diabetic animals were observed with dyslipidemia. Oral administration of novel triterpenoids reduced the serum TC, TG, LDL and VLDL and increased HDL levels. In STZ-administered diabetic animals, the activity of glucokinase was reduced; treatment with the novel compounds improved the entry of glucose into the cells, which may in turn have stimulated the production of this enzyme. Glucose-6-phosphatase is an important regulatory enzyme in gluconeogenesis. The amount of enzyme has been shown to be increased in diabetic animals [28], as also reported in the present investigation. However, oral administration of novel compounds reduced glucose-6-phosphatase activity, indicating that the novel compounds inhibited gluconeogenesis in the liver. Asiatic acid, a triterpenoid derivative of Centella asiatica, improved glucose-6-phosphatase, fructose-1 and 6-bisphosphatase of carbohydrate metabolism in STZ-induced diabetes rats [29]. In our study, there was a concomitant increase in glycogen synthase with glycogen phosphorylase enzyme activity caused by the reduced level of insulin in the diabetic state, which resulted in the glycogen synthetase system’s inactivation. The altered levels of these enzymes were reestablished in compound-treated diabetic rats. Glucose oxidation is a critical mechanism that provides energy to the cells to perform different functions. The rate of glucose oxidation in a cell is proportional to the glucose entering the cell [30]. The decreased oxidation rate in the skeletal muscle of diabetic rats may be attributed to decreased GLUT4 levels that lead to elevated blood glucose.

Deficient post receptor insulin signaling is the key characteristic involved in insulin resistance [31]. IR is the primary metabolic transition in the insulin-signaling pathway needed for the translocation of GLUT4 molecules to the plasma membrane. The activated IR phosphorylates several proteins, such as IRS-1, -2, -3 and -4. Therefore, the efficacies of novel triterpenoids were assessed in regard to IR and IRS-1 mRNA and protein expression. Diabetic rats showed decreased IR and IRS-1 mRNA and protein levels, thereby providing a possible molecular explanation for the decreased insulin sensitivity, while treatment with novel triterpenoids (C1–C3) showed a substantial increase IRand IRS-1 expression.

Akt activation involves phosphorylation of both threonine and serine residues. Data from animal studies have suggested that insulin resistance developed viad efects in both upstream and downstream Akt/PKB targets in the form of dephosphorylation of protein side chains or complete loss of IRS proteins, with reduced PI3K activity and impaired Akt/PKB phosphorylation substrates [32]. In the present study, administration of the novel triterpenoids(C1–C3) improved the expression of Akt mRNA, Akt protein and its serine phosphorylation to levels equal to those innormal control and insulin-treated models.

GLUT4 is the main transporter that mediates the absorption of glucose into insulin-sensitive skeletal muscle and adipose tissue [33]. Vesicles containing GLUT4 are translocated from the cytoplasm to the plasma membrane after the binding of insulin to its receptor [34]. Decreased translocation of GLUT4 is a cause of insulin resistance in diabetes [35]. In STZ-administered diabetic animals, the expression of GLUT4 mRNA and protein was reduced significantly, while compound administration to diabetic rats increased the same. In this regard, our laboratory study showed that the administration of catechin isolated from C. fistula to diabetic rats upregulated the expression of GLUT4 mRNA and protein. Similar studies revealed the hypoglycemic effects of cucurbitane triterpenoid isolated from bitter melon on GLUT4 translocation in the L6 muscle cells and 3T3-L1 adipocytes though activation of the AMPK pathway, as well as those of triterpenes isolated from Lamiaceae plant species in 3T3-L1 adipocytes through activation of the PI3K pathway [36,37].

Molecular docking helped to discover an appropriate ligand for the receptor by generating an optimal protein–ligand bound conformation. All docking results were monitored by scoring functions that predicted the binding affinity of a ligand in a particular docked pose [38]. High glide scores indicated good binding between a ligand and receptor [39]. The results of the present study showed that the selected compounds obtained good docking scores and receptor–ligand binding affinity. Glide energy also played a vital role in validating the docking results; minimum glide energy indicated that a ligand was buried in the cavity of the receptor [40]. In the present study, all the compounds produced minimum glide scores with the target proteins, which revealed that these compounds bound well in the binding cavities of the receptors. Hydrogen bonding interactions also show that the interactions of compounds are made with active site residues of the target proteins. Here, we identified that compound **1** had two H bond interactions with IRK protein. The H-O group of the ligand molecule interacted with the NH groups of two different amino acids, namely SER 1090 and ALA 1080. Compound **2** had hydrogen bonding interactions with GLN 1004 (OH…O=C) and PHE 1151 (NH…O=C), whereas compound **3** had three H bond interactions, with ALA 1080 (OH…O=C), PHE 1151 (NH…O=C) and ASP 1083 (OH…O=C). PPAR-γ also showed hydrogen bonding interactions with all three compounds. The OH group of compound **1** interacted with the NH group of the amino acid GLN 283. The OH group of compound **2** had two H bond interactions, with ASP 260 (OH…O=C) and SER 342 (OH…O=C), and compound **3** had two H bond interaction, with 260 (OH…O=C) and GLU 343 (OH… O=C).

## 4. Materials and Methods

### 4.1. Plant Material

During summer, fresh bark of C. fistula was collected from Kodaikanal, Tamil Nadu, India. The species was identified and authenticated at the Department of Botany, Holy Cross College, Tiruchirapalli, and the voucher specimen was deposited in the herbarium of the Department.

### 4.2. Isolation and Identification of Compounds

The shade-dried plants were powdered, and 1 kg of powder was extracted sequentially using hexane, ethyl acetate and methanol in a Soxhlet apparatus. The extracts were evaporated to dryness under reduced pressure in a rotary evaporator. The yield of hexane extract was 17.8 g, that of ethyl acetate extract was 16.6 g and that of methanol extract was 20.1 g. The dry residues of these crude extracts were stored at 4 °C for further use. The ethyl acetate extract was further chromatographed on a silica gel column (Merck 70–230 mesh, 400 g, 3.5 i.d. × 60 cm) and then successively eluted with a continuous gradient from 100% hexane, 95% hexane and 5% ethyl acetate to10% ethyl acetate. The fractions were collected, and each fraction was spotted on a precoated Silica gel 60 F254, 0.25 mm thick thin layer chromatography (TLC) plate (Merck). Fractions with similar Rf values in the TLC pattern were pooled together into 18 fractions. Fractions 2, 8 and 9 gave single spots in TLC. The compounds were subjected to nuclear magnetic resonance (NMR) and mass (MS) and infrared (IR) spectral analyses [41] for structural determination.

### 4.3. In Vivo Analysis

#### 4.3.1. Animals

Healthy adult male albino Wistar rats (weighing 150–220 g) were used in the present study. Animals were maintained under specific temperature (21 ± 2 °C) and humidity (65 ± 5%) with a constant 12 h light and 12 h dark schedule and fed with a standard pelleted diet (Lipton India, Mumbai, India) and clean drinking water ad libitum.

#### 4.3.2. Induction of Experimental Diabetes

In the experimental animals, after overnight fasting, diabetes was induced by single intraperitoneal injection of60 mg/kg body weight STZ [42] in a freshly prepared citrate buffer (0.1 M, pH 4.5). In order to avoid initial drug-induced hypoglycemic mortality, 20% glucose solution was fed to STZ-injected rats for 24 h. After few days, the STZ-administered rats developed severe glycosuria and hyperglycemia. Diabetes induction was confirmed by assessing the level of fasting blood glucose 96 h after STZ administration. The rats that showed blood glucose concentrations of ˃250–400 mg/dL were considered diabetic and used for further experimentation.

#### 4.3.3. Experimental Design

In this study, a total of 54 animals were divided into 9 groups of 6 animals each. Group 1: vehicle control (normal control animals treated with 0.5% carboxy methyl cellulose (CMC), 1 mL/kg body weight); Groups 2–4: normal control rats treated with novel triterpenoids (20 mg/kg body weight); Group 5: STZ-induced diabetic rats; Groups 6–8: diabetic rats treated with novel triterpenoids (20 mg/kg body weight); Group 9: insulin-treated diabetic animals (3 IU/kg body weight). The dosage of compounds was fixed from the acute toxicity tests reported in our previous study, which were filed for patent [43,44,45]. The compounds were dissolved in vehicle solution and orally administered daily using an intragastric intubation for 60 days with a periodical estimation of blood glucose level (i.e., on the 0th, 7th, 15th, 30th, 45th and 60th days). At the end of the experimental duration, the animals were fasted overnight, anesthetized with IP ketamine (75 mg/kg) and xylazine (10 mg/kg) and sacrificed by cervical dislocation. Blood was collected, sera and plasma were isolated and liver and skeletal muscle from control, diabetic and treated animals were dissected out and stored at −80 °C for further analysis.

### 4.4. Determination of Blood Glucose

Serum blood glucose concentration was determined by a commercially available glucose kit (CPC diagnostics, Barcelona, Spain). Results were expressed as mg/dL.

### 4.5. Measurement of Plasma Insulin and C-Peptide

Radioimmunoassay was used to test plasma insulin using a kit method (Diasorin, Saluggia, Italy). The kit included human insulin as standard and ^125^I-labelled human insulin antibody, which has cross-reactivity with rat insulin. Plasma C-peptide level was assayed by a radioimmunoassay kit procured from Missouri, USA. Results were expressed as µmol/mL.

### 4.6. Assessment of Tissue Glycogen

Glycogen was estimated in the liver and skeletal muscle of the control and treated animals [46]. Five milligrams of tissue was digested with 1 mL of 30% KOH for 20 min in a boiling water bath. The contents were cooled on an ice bath, and 1.25 mL of 95% ethanol was added, thoroughly mixed and gently brought to boil in a hot water bath, then cooled and centrifuged for 15 min at 3000× *g*. The supernatant was decanted, and the tubes were allowed to drain on a filter paper for few minutes. The precipitate was dissolved in 1 mL of distilled water, precipitated with 1 mL of 95% ethanol, centrifuged and drained as stated before. The precipitate was dissolved in 5 mL of distilled water, and 10 mL of 0.2% anthrone reagent was added under ice-cold conditions. Five milliliters of distilled water and a series of standards with final volumes of 5 mL were treated with the anthrone reagent and subjected to the same procedure. The tubes were covered with glass marbles and heated for 10 min in a boiling water bath. The contents were cooled immediately, and the color developed was read at 680 nm. The amount of glycogen was expressed as g/100 g of wet tissue.

### 4.7. Estimation of Enzymes of Carbohydrate Metabolism

According to the standard methods [47], glucokinase and glucose-6-phosphatase were analyzed. Results were expressed as Pi mmol liberated/h/protein. The standard methods were used to estimate glycogen synthase and glycogen phosphorylase. Results were expressed as UDP-formed mmol/h/protein.

### 4.8. Assessment of Lipid Markers

Serum TC, TG, HDL and LDL were measured as per the manufacturer’s instructionsusing akit procured from Diagnostic kit (Beacon Diagnostics, Kabilpore, Navsari, India). Results were expressed as mg/dL.

### 4.9. Estimation of Glucose Oxidation

Oxidation of the glucose in the skeletal muscle of the control and treated animals was measured [48] using ^14^C-glucose. Briefly, 10 mg tissue was kept in a 2 mL ampoule containing 170 µL Dulbecco’s modified Eagle’s medium (DMEM), pH 7.4, 10IU penicillin and 0.5 μCi ^14^C-glucose. The ampoules were then aerated with a CO_2_trap containing a gas mixture. The closed system with the CO_2_ trap was then kept in an incubator at 37 °C. The CO_2_trap was replaced after 2 h. After removing the second trap and releasing any remaining CO_2_ from the sample, 0.1 mL of NH_2_SO_4_ was applied to prevent further metabolism. The process was closed 1 h before the third once again, and the final trap was removed. Finally, all the CO_2_ traps were placed in a scintillation vial containing10 mL scintillation fluid and counted in a β-counter. Results of ^14^CO_2_ released/10 mg tissue were expressed as CPM.

### 4.10. Gene expression Analysis

#### Isolation of Total RNA, Conversion of cDNA and Analysis of Real-Time PCR

Total RNA was isolated using a TRIR kit (Total RNA Isolation Reagent, Invitrogen). Briefly, 1 mL TRIR was added to 100 mg of tissue and homogenized. The contents were transferred into a microcentrifuge tube, and 0.2 mL chloroform was added, vortexed for 1 min and kept at 4 °C for 5 min, then centrifuged at 12,000× *g* for 15 min at 4 °C. The aqueous phase (upper layer) was carefully transferred to a fresh microcentrifuge tube, and isopropanol was added in equal quantity, vortexed for 15 s and placed on ice for 10 min. Then, the contents were centrifuged for 10 min at 4 °C at 12,000× *g*, the supernatant was discarded, and the RNA pellets were washed by quick vortexing with 1 mL of 75% ethanol. The total RNA was quantified using a spectrophotometer procedure [49]. The total RNA concentrations were expressed in micrograms (μg). The purity of the total RNA was 1.7 to 1.9. Using the Eurogentec (Seraing, Belgium) reverse transcriptase kit, complementary DNA (cDNA) was synthesized from 2μg total RNA, as specified in the manufacturer’s protocol. Real-timePCR was then performed [50]. Briefly, areaction mixture containing 2 × reaction buffer (Takara SyBr green master mix), the forward and reverse primers of the target gene and household gene and water (the primer sequences are listed in Table 2) with a total volume of 45 μL was made, thoroughly mixed and spun. Five microlitersof control DNA for positive control, 5 μL of water for negative control and 5 μL of template cDNA for samples were kept in separate PCR vials, and reaction mixture (45 μL) was added. The reaction was set up with 40 cycles (95 °C for 5 min, 95 °C for 5 s, 60 °C for 20 s and 72 °C for 40 s), and the results obtained were plotted by the PCR machine (Stratagene MX 3000 P, Agilent Technologies, 530 l, Stevens Creek Blvd, Santa Clara, CA, USA) on a graph. Relative quantification was calculated from the melt and amplification curve analysis.

### 4.11. Protein Expression Analysis

#### 4.11.1. Preparation of Sample

Tissue samples from the gastrocnemius muscle were prepared as per the standard protocol [54]. Then, the concentration of proteins was determined [55] before a Western blot analysis was performed.

#### 4.11.2. Western Blot Analysis

Lysate proteins (50 μg/lane) were separated using 10% sodium dodecyl sulfate–polyacrylamide gel electrophoresis (SDS–PAGE) and transferred to a membrane by electroblotting (Bio-Rad Laboratories Inc., Hercules, CA, USA) of polyvinylidene difluoride (PVDF). The membrane was blocked and tested with primary antibodies of IR-β, IRS-1, p-IRS-1^Tyr612^, Akt 1/2/3 and p-Akt^Ser473^, GLUT4 (polyclonal antibodies were purchased from Santa Cruz Biotechnology, Inc., Dallas, TX, USA) and β-actin monoclonal (at 1:1000 dilution) using 5% nonfat dried milk.

Upon incubation the membrane was washed with tris-buffered saline containing Tween20 (TBS-T) three times (5 min each). Then, washed with TBS-T, horseradish peroxidase (HRP)-conjugated rabbit-anti-mouse or goat-anti-rabbit antibodies (at 1:5000, GeNei, Bangalore, India) were incubated with the membrane for 1 h. Using an enhanced chemiluminescence detection system (Thermo Fisher Scientific Inc., Waltham, MA, USA), the specific signals were detected, and using Chemidoc, the protein bands were captured and quantified (Bio-Rad Laboratories Inc., Hercules, CA, USA Quantity, One image analysis method). Afterward, the membrane was reprobed with β-actin antibody (at 1:5000). In the present study, rat β-actin was used as a loading control.

### 4.12. Statistical Analysis

Statistical analysis was conducted using the SPSS software package version 17.0. The values were evaluated using a one-way variance analysis (ANOVA) followed by the multiple-range test (DMRT) [56].

### 4.13. In Silico Analysis

#### 4.13.1. Protein Preparation

The crystal structures of IRK and PPAR-γ were downloaded from PDB (http://www.rcsb.org, accessed on 25 October 2021) with the accession IDs1IRK and 1ZGY, respectively. Two types of facilities (i.e., preparation and refinement) were accessible in the “protein preparation wizard”of the Schrodinger software (Maestro, version 9.3, Schrodinger, LLC, New York, NY, USA, 2012). First, the chemical accurateness was ensured; then, hydrogen atoms were added, and side chains that were neither near the active site nor participatoryin the creation of salt bridges were neutralized by using the optimized potentials for liquid simulations–all atom (OPLS–AA) force field (protein preparation wizard, Schrodinger, LLC, New York, 2014).

#### 4.13.2. Ligand Preparation

The three novel compounds were drawn in Maestro and allocated structures using the LigPrep package from Schrodinger, LLC, New York (LigPrep (2.4) Schrodinger, LLC. New York). These structures were changed to the.mae format, suitable bond orders were given for every structure and tautomers for all of these ligands were produced and optimized. Partial atomic charges were computed using the OPLS2005 force field.

#### 4.13.3. Molecular docking

The three selected novel compounds were docked into the active sites of IRK and PPAR-γ using the Glide XP module (Glide, version 6.2, Schrodinger, LLC, New York, 2014). The shapes and properties of the target proteins were shown on a grid by many sets of fields that gradually gave more perfect scoring to the ligand poses. The grid wassetto 12 Å × 12 × Å 12 × Å for the analysis of docking study. Geometric or hydrogen-bonding restraints were not introduced for substrate docking. Docking studies were carried out with the default parameters.

## 5. Conclusions

Our present findings clearly showed that the novel triterpenoid compounds from *C. fistula* controlled hyperglycemia through activation of IRS-1/Akt-mediated insulin signaling mechanisms and through regulation of carbohydrate metabolic enzymes in the skeletal muscle and liver. Hence, we concluded that supplementation of triterpenoids could be a promising therapeutic approach in the management of diabetes. Clinical trials engaging such triterpenoids could be of intensive interest and beneficial for the management of diabetes.

## Figures and Tables

**Table 1:** Glide score and glide energy of 3 novel compounds with 1-IRK and PPAR-γ proteins.

Compound	Receptor	Docking Score (kcal/mol)	Docking Energy (kcal/mol)	H-Bond
Novel compound **1**	1-IRK	−10.57	−106.28	SER 1090 ALA 1080
Novel compound **2**	1-IRK	−10.36	−98.84	GLN 1004 PHE 1151
Novel compound **3**	1-IRK	−10.21	−98.01	ALA 1080 PHE 1151 ASP 1083
Novel compound **1**	PPAR-γ	−10.81	−103.72	GLN 283
Novel compound **2**	PPAR-γ	−10.46	−95.40	ASP 260 SER 342
Novel compound **3**	PPAR-γ	−10.01	−96.62	ASP 260 GLU 343

**Table 2:** Details of primer sequences.

Name of Gene	Primer Sequence	Amplicon Size	Reference
Rat IR	FW: 5′-GCC ATC CCG AAA GCG AAG ATC-3’ RW: 5′-TCT GGG TCC TGA TTG CAT-3’	224 bp	[51]
Rat IRS-1	FW: 5’-GCC AAT CTT CAT CCA GTT GCT-3’ RW: 5’-CAT CGT GAA GAA GGC ATA GGG-3’	336 bp	[51]
Rat Akt	FW: 5′-GGA AGC CTT CAG TTT GGA TCC CAA-3′ RW: 5′-AGT GGA AAT CCA GTT CCG AGC TTG-3′	146bp	NM_017093.1
Rat GLUT4	FW: 5′-GGG CTG TGA GTG AGT GCT TTC-3′ RW: 5′-CAG CGA GGC AAG GCT AGA-3′	150 bp	[52]
Rat β-actin	FW: 5′-AAG TCC CTC ACC CTC CCA AAA G-3’ RW: 5′-AAG CAA TGC TGT CAC CTT CCC-3’	374 bp	[53]

## Data Availability

Not applicable.

## Supplementary Materials

Figure S1: Spectral analysis of novel compound **1**, Figure S2: Spectral analysis of novel compound **1**, Figure S3: Spectral analysis of novel compound **1**, (a) IR spectrum; (b) mass spectrum; (c) ^1^H-NMR spectrum; (d) ^13^C-NMR spectrum.
