# Supplementary material for: Novel Triterpenoids from Cassia fistula Stem Bark Depreciates STZ-Induced Detrimental Changes in IRS-1/Akt-Mediated Insulin Signaling Mechanisms in Type-1 Diabetic Rats
Source: Molecules. 2021 Nov 11;26(22):6812. doi: 10.3390/molecules26226812 (PMC8621110; doi:10.3390/molecules26226812)
Supplement: Supplementary file 1 [file molecules-26-06812-s001.zip › molecules-1452084-supplementary.pdf]

molecules

Supplementary File

Novel Triterpenoids from *Cassia fistula* Stem Bark Depreciates STZ-Induced Detrimental Changes in IRS-1/Akt-Mediated Insulin Signaling Mechanisms in Type-1 Diabetic Rats

SabapathyIndu [1], PeriyasamyVijayalakshmI [1], JayaramanSelvaraj [2] and ManikkamRajalakshmI [1,*]

[1] DBT-BIF Centre, PG & Research Department of Biotechnology & Bioinformatics, Holy Cross College (Autonomous),Bharathidasan University, Trichy 620002, Tamil Nadu, India; sabaindhu2010@gmail.com (S.I.); pvijibi@gmail.com (P.V.)
[2] Department of Biochemistry, Saveetha Institute of Medical and Technical Sciences,Saveetha Dental College and Hospitals, Saveetha University, Chennai 600020, Tamil Nadu, India; jselvaendo@gmail.com
* Correspondence: rajalakshmi@hcctrichy.ac.in; Tel.: +91–99524 98098

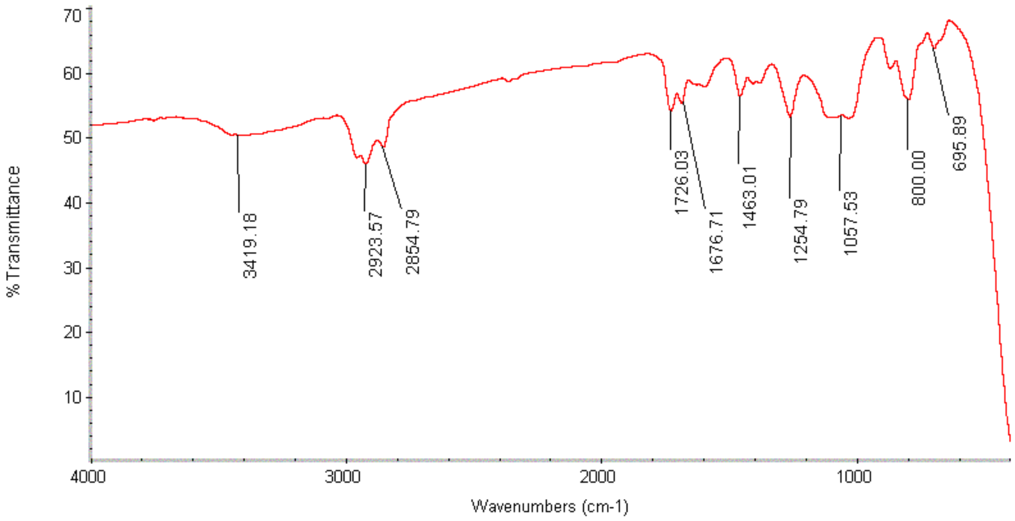
% Transmittance
70
60
50
40
30
20
10
3419.18
2923.57
2854.79
1726.03
1676.71
1463.01
1254.79
1057.53
800.00
695.89
4000
3000
2000
1000
Wavenumbers (cm-1)

(**a**)

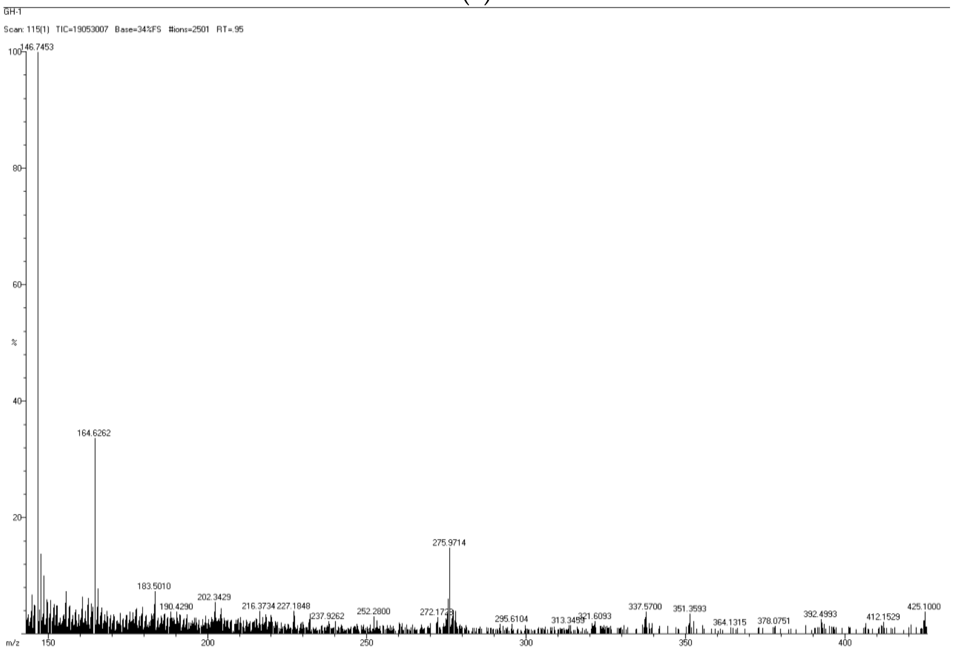
GH-1
Scan: 115(1) TIC=19053007 Base=34%FS #ions=2501 RT=.95
100
80
60
%
40
20
146.7453
164.6262
183.5010
190.4290
202.3429
216.3734
227.1848
237.9262
252.2800
275.9714
295.6104
321.6093
337.5700
351.3593
364.1315
378.0751
392.4993
412.1529
425.1000
m/z
150
200
250
300
350
400

(**b**)

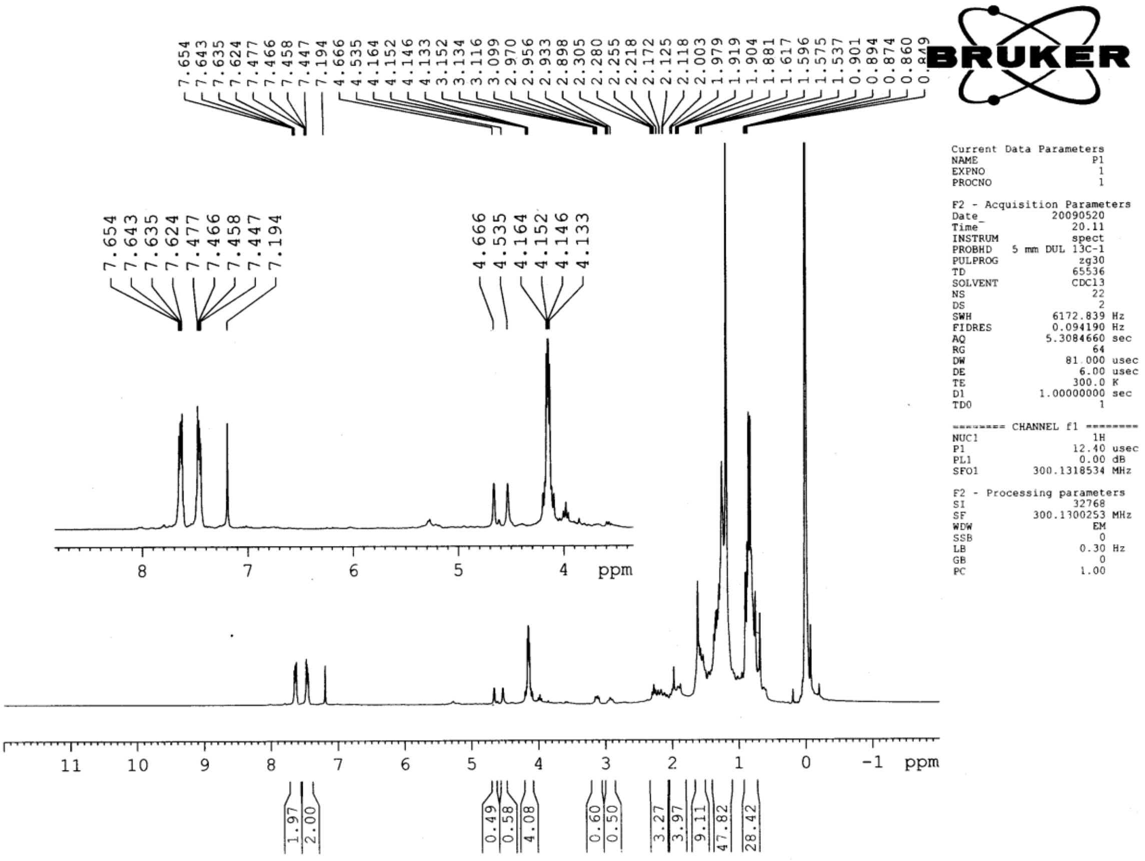

(**c**)

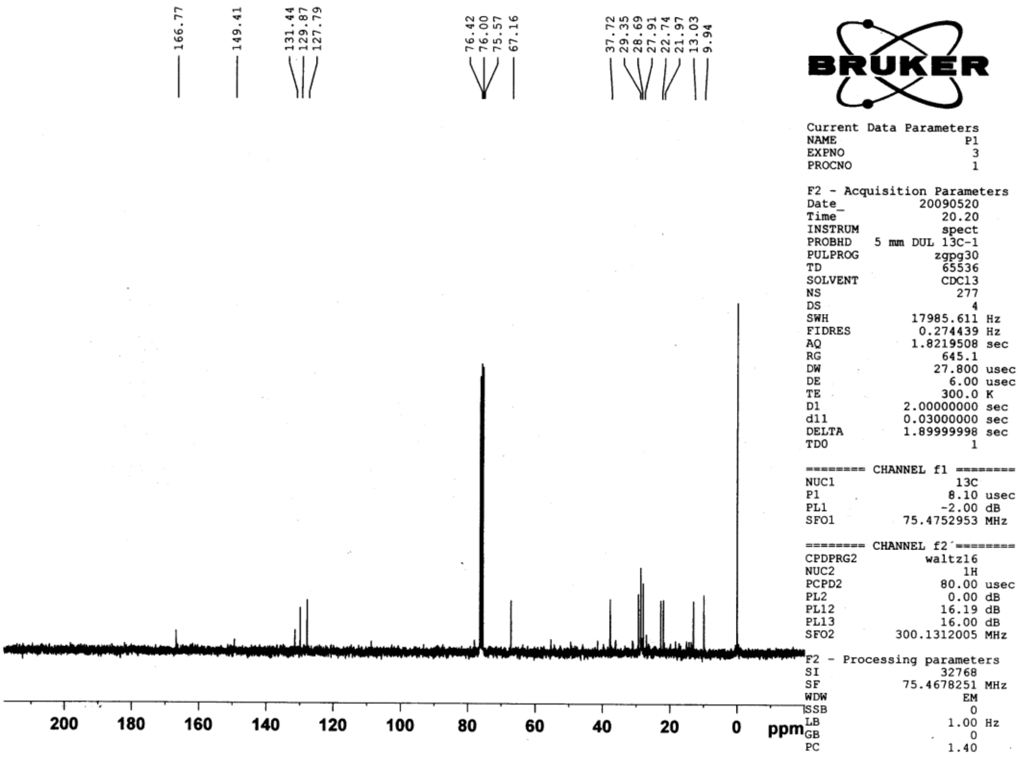

(**d**)

Figure S1. (**a**): IR- spectrum of Novel triterpenoid Cpd-I. (**b**): Mass spectrum of Novel triterpenoid Cpd-I. (**c**): ^{1}H-NMR spectrum of Novel triterpenoid Cpd-I. (**d**): ^{13}C-NMR spectrum of Novel triterpenoid Cpd-I.

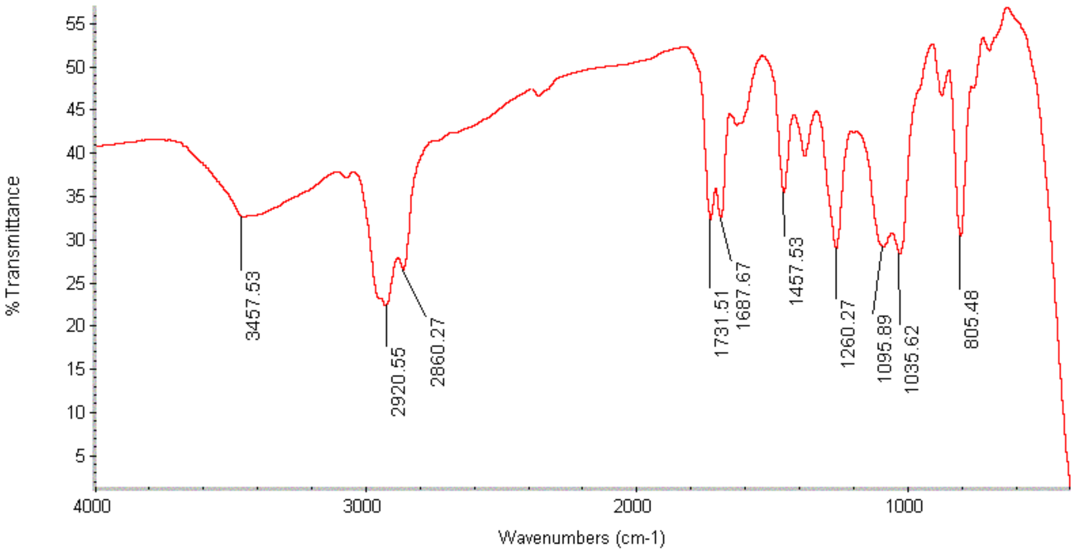
% Transmittance
Wavenumbers (cm-1)
3457.53
2920.55
2860.27
1731.51
1687.67
1457.53
1260.27
1095.89
1035.62
805.48

(**a**)

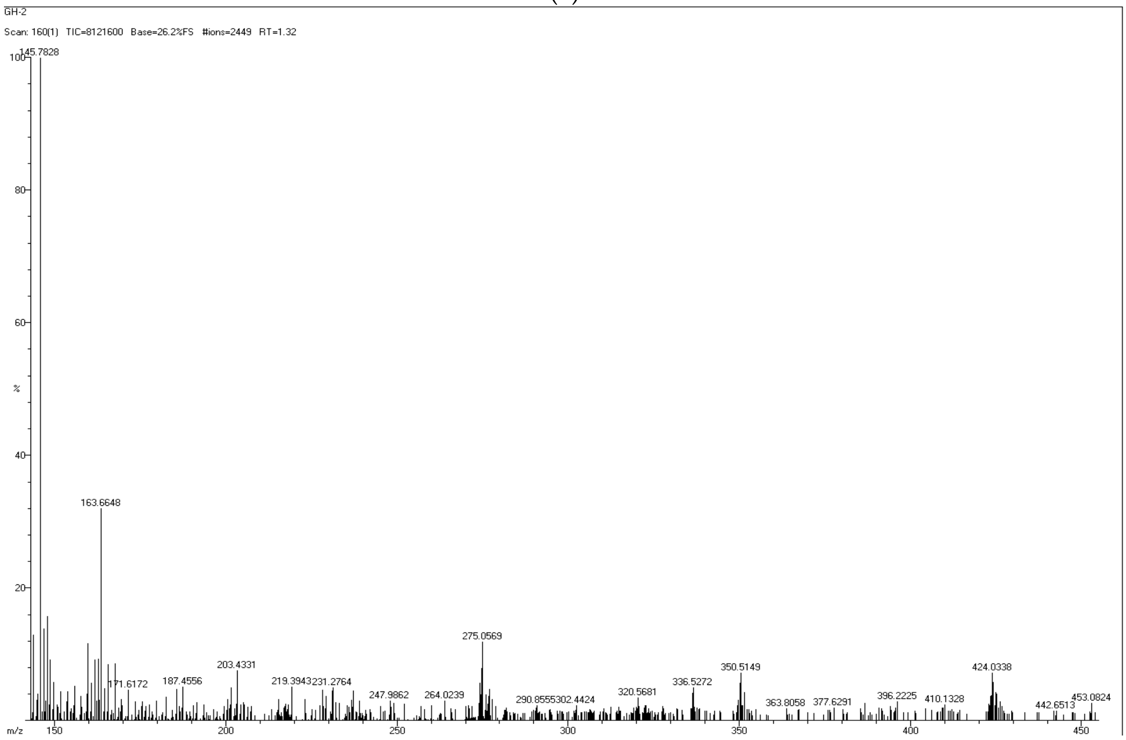
GH-2
Scan: 160(1) TIC=8121600 Base=26.2%FS #ions=2449 RT=1.32
145.7828
163.6648
171.6172
187.4556
203.4331
219.3943
231.2764
247.9862
264.0239
275.0569
290.8555
302.4424
320.5681
336.5272
350.5149
363.8058
377.6291
396.2225
410.1328
424.0338
442.6513
453.0824
m/z
%

(**b**)

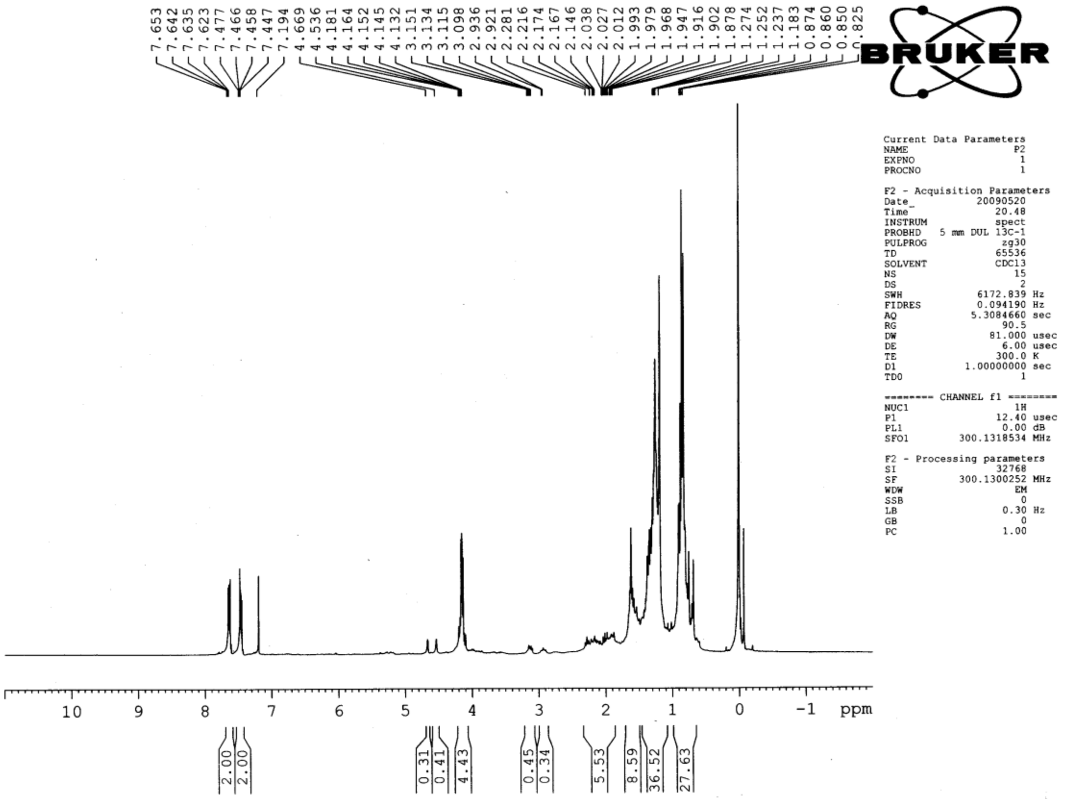

(**c**)

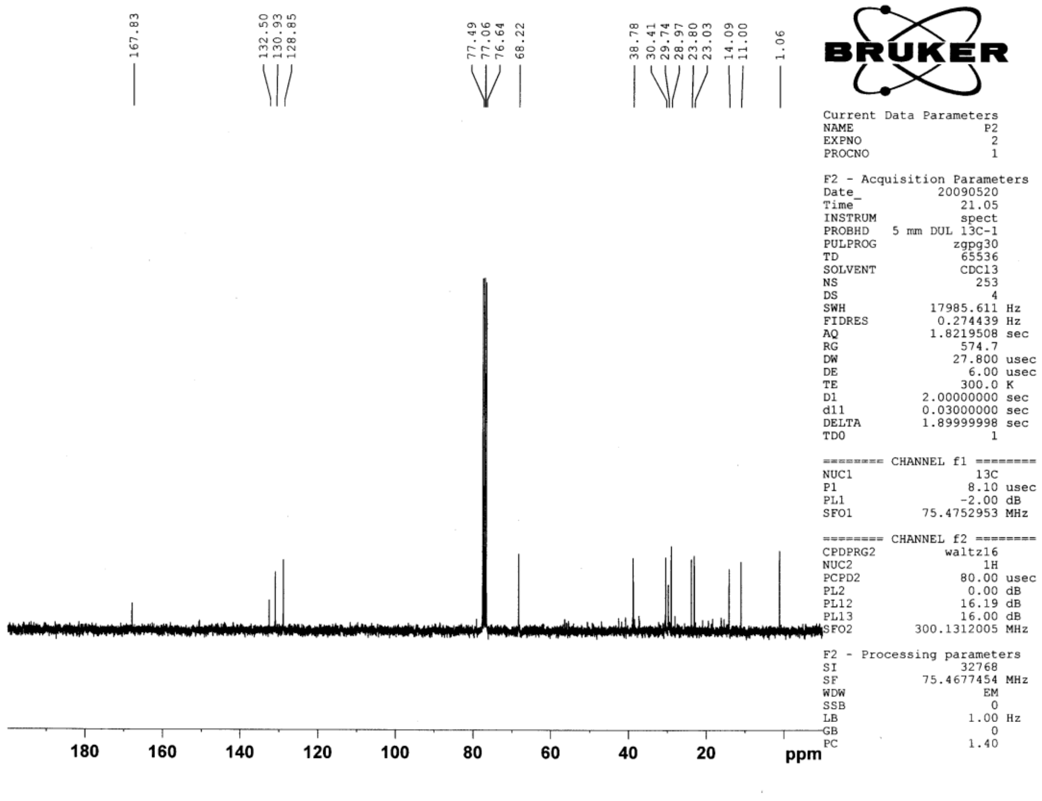

(**d**)

Figure S2. (**a**): IR- spectrum of Novel triterpenoid Cpd-II. (**b**): Mass spectrum of Novel triterpenoid Cpd-II. (**c**): ^{1}H-NMR spectrum of Novel triterpenoid Cpd-II. (**d**): ^{13}C-NMR spectrum of Novel triterpenoid Cpd-II.

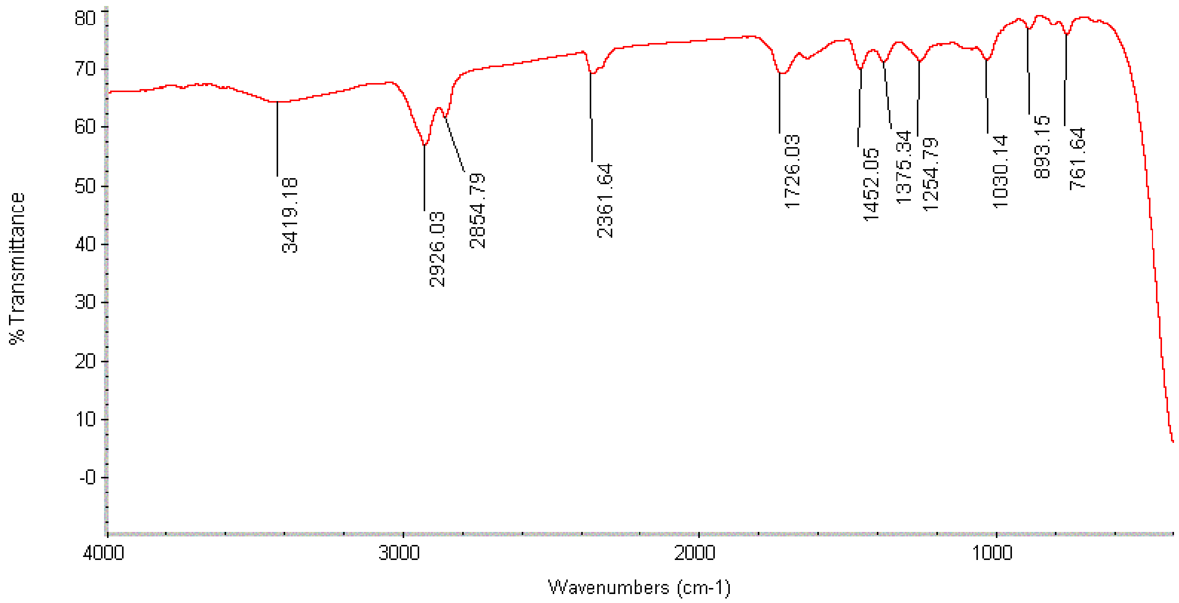
% Transmittance
Wavenumbers (cm-1)
3419.18
2926.03
2854.79
2361.64
1726.03
1452.05
1375.34
1254.79
1030.14
893.15
761.64

(**a**)

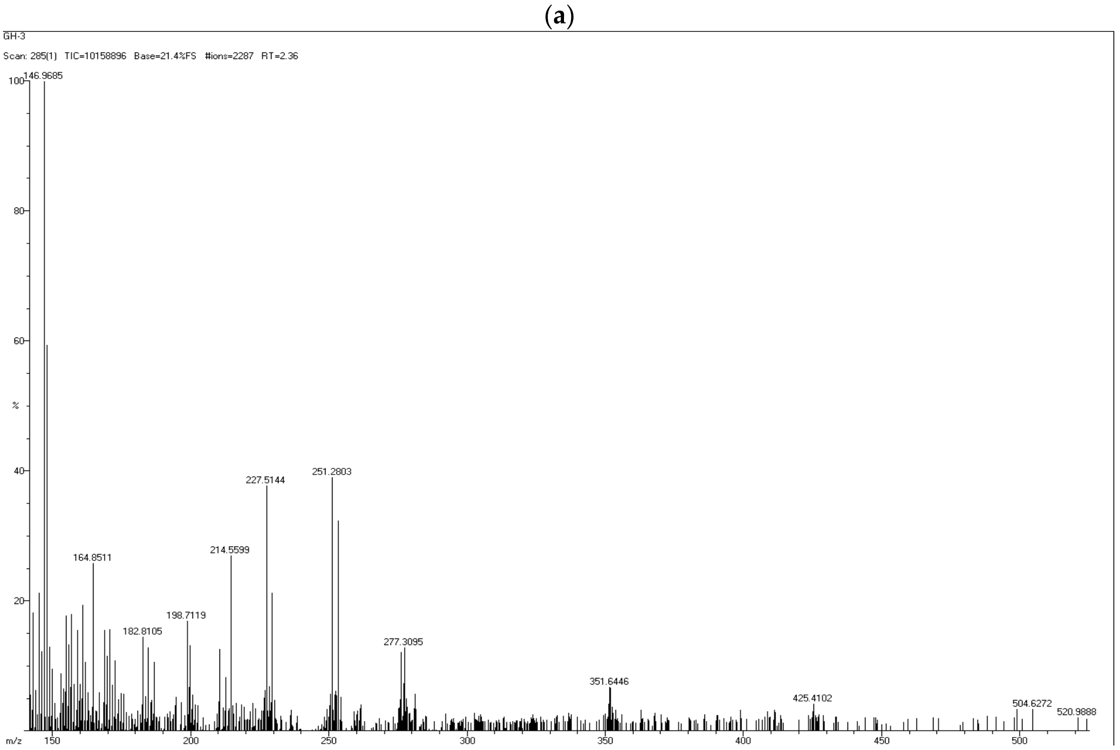
GH-3
Scan: 285(1) TIC=10158896 Base=21.4%FS #ions=2287 RT=2.36
146.9685
164.8511
182.8105
198.7119
214.5599
227.5144
251.2803
277.3095
351.6446
425.4102
504.6272
520.9888
%
m/z

(**b**)

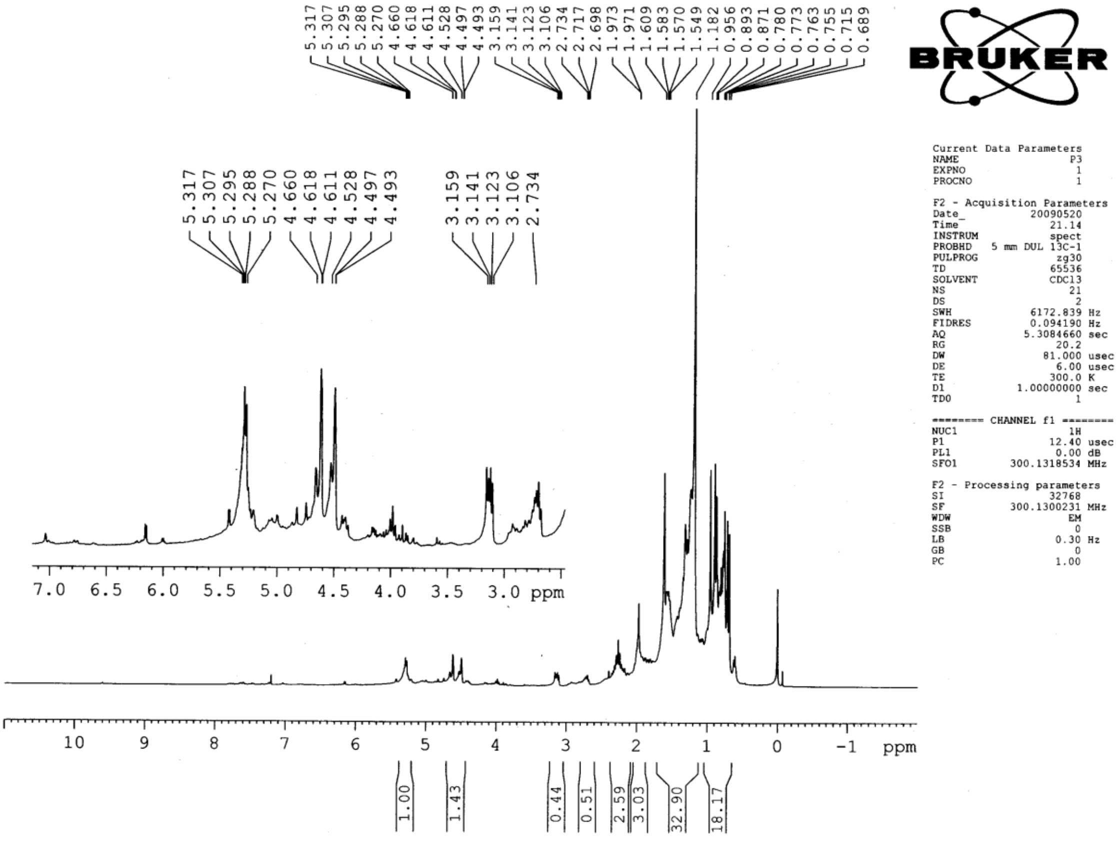

(**c**)

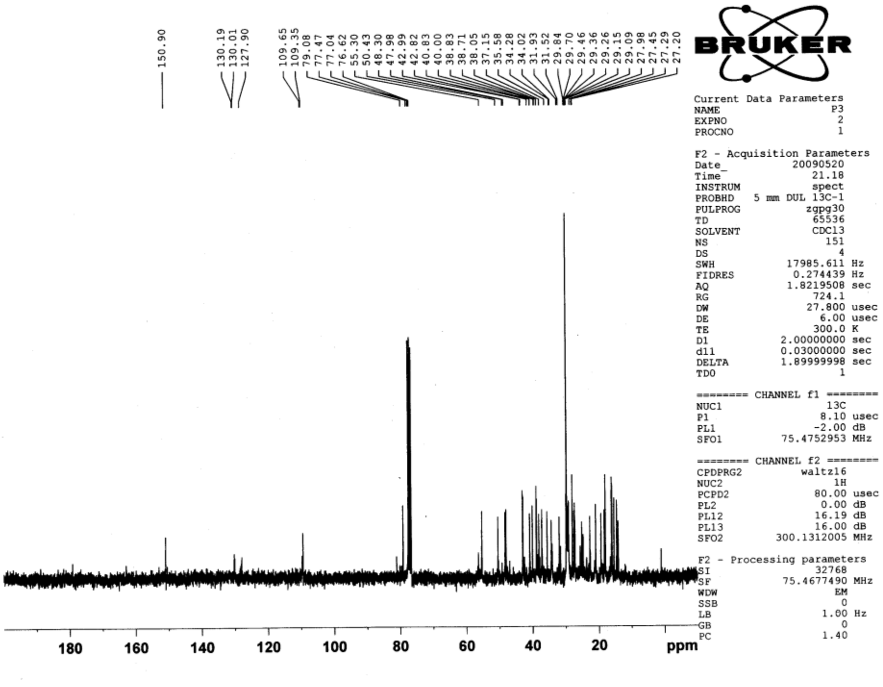

(**d**)

Figure S3. (**a**): IR- spectrum of Novel triterpenoid Cpd-III. (**b**): Mass spectrum of Novel triterpenoid Cpd-III. (**c**): ^{1}H-NMR spectrum of Novel triterpenoid Cpd-III. (**d**): ^{13}C-NMR spectrum of Novel triterpenoid Cpd-III.
